# The personality and cognitive traits associated with adolescents’ sensitivity to social norms

**DOI:** 10.1038/s41598-022-18829-x

**Published:** 2022-09-09

**Authors:** Christopher Tate, Rajnish Kumar, Jennifer M. Murray, Sharon Sanchez-Franco, Olga L. Sarmiento, Shannon C. Montgomery, Huiyu Zhou, Abhijit Ramalingam, Erin Krupka, Erik Kimbrough, Frank Kee, Ruth F. Hunter

**Affiliations:** 1 School of Medicine, Dentistry and Biomedical Sciences, Centre for Public Health, Institute of Clinical Sciences Block B, Royal Victoria Hospital, Queen’s University Belfast, Grosvenor Road, Belfast, BT12 6BA UK; 2 Queen’s Management School, Queen’s University Belfast, Riddel Hall, 185 Stranmillis Road, Belfast, BT9 5EE UK; 3 Department of Public Health, School of Medicine, Universidad de Los Andes, Carrera 1 No 18 A-10, Bloque Q Piso 8, Postal Code: 57 Bogotá, Colombia; 4 Department of Family and Child Sciences, Florida State University, Sandels Building, 675 W Call St, Tallahassee, FL 32304 USA; 5 F3 Informatics Building, School of Computing and Mathematical Sciences, University of Leicester, University Road, Leicester, LE1 7RH UK; 6 Department of Economics, Appalachian State University, 416 Howard Street, Peacock Hall 3101A, ASU Box 32051, Boone, NC 28608 USA; 7 School of Information, University of Michigan, 105 S State St, Ann Arbor, MI 48109 USA; 8 Smith Institute for Political Economy and Philosophy, Chapman University, 1 University Dr, Orange, CA 92866 USA

**Keywords:** Human behaviour, Personality

## Abstract

Little is known about the personality and cognitive traits that shape adolescents’ sensitivity to social norms. Further, few studies have harnessed novel empirical tools to elicit sensitivity to social norms among adolescent populations. This paper examines the association between sensitivity to norms and various personality and cognitive traits using an incentivised rule-following task grounded in Game Theory. Cross-sectional data were obtained from 1274 adolescents. Self-administered questionnaires were used to measure personality traits as well as other psychosocial characteristics. Incentivised rule-following experiments gauged sensitivity to social norms. A series of multilevel mixed effects ordered logistic regression models were employed to assess the association between sensitivity to norms and the personality and cognitive traits. The results highlighted statistically significant univariate associations between the personality and cognitive traits and sensitivity to norms. However, in the multivariate adjusted model, the only factor associated with sensitivity to norms was gender. The gender-stratified analyses revealed differences in the personality and cognitive traits associated with sensitivity to norms across genders. For males need to belong was significantly negatively associated with sensitivity to norms in the multivariate model. By comparison, emotional stability was negatively associated with sensitivity to norms for females. This study reinforced the findings from an earlier study and suggested female adolescents had higher levels of sensitivity to norms. The results indicated no consistent pattern between sensitivity to norms and the personality and cognitive traits. Our findings provide a basis for further empirical research on a relatively nascent construct, and bring a fresh perspective to the question of norm-following preferences among this age group.

## Introduction

Adolescence is a developmental period characterised by a heightened sensitivity to social influences that engender behavioural change. The social environment takes on greater significance during the transition to adulthood as the influence of others becomes more salient^1^. Many researchers have attempted to delineate the complex cognitive processes involved in navigating the social environment during adolescence. A review of research on adolescent social cognitive development^2^ highlighted the importance of sensitivity to socio-environmental cues and the role of social acceptance in adolescent decision-making. The social reorientation that occurs during adolescence is marked by a progressive transition away from the proximal family setting in favour of the peer group context^3^. Therefore, the extent to which an adolescent is sensitive to norms that compel them to conform to the behaviours of others is an important consideration for health behaviour research. Broadly defined, “sensitivity to norms” is an individual’s propensity to adhere to a social norm. In this paper, our focus is narrowed to include injunctive norms, which are the perceived expectations to conform to a behaviour and the actions an individual believes they ought to take to avoid social sanctions^4^.

The increase in risk-taking during adolescence has been attributed to the need to avoid social sanctions from peers^5^. Thus, it can be argued that social behaviour is driven by motivations to integrate with peers^6^. There are numerous studies that examine peer influence and peer selection effects^7^. Prinstein et al.^8^ demonstrate that the influence of high-status peers on adolescents’ deviant behaviour is associated with higher levels of susceptibility. Further, Teunissen et al.^9^ showed that susceptibility to pro-alcohol norms of popular peers moderated the relationship between perceived friends’ drinking norms and adolescents’ alcohol use. In a recent review, Do et al.^10^ used Differential Susceptibility Theory to identify neurobiological determinants of susceptibility to social influence. The authors note that “while person- and environment-specific factors independently affect susceptibility to social influence in adolescence, examining interactions between these characteristics is critical for identifying those who may be most susceptible, which can have both adaptive and maladaptive developmental outcomes” (p. 8).

The influence of norms can be captured in a simple “norm-dependent” utility function in which individuals care about both consumption utility and injunctive norms of behaviour, which depend on the choice setting. Understanding how an individual’s choices vary as a function of the disutility they experience by violating a given norm can provide valuable insight into behaviour in social settings. For example, if it is known that smoking is a desirable social trait among a particular friend group, an individual will increase his/her utility by smoking. Note that the utility function treats an increase in esteem from choosing to smoke and a reduction of social sanctions that would be incurred from choosing not to smoke in the same way; if an individual smokes, we can infer that the utility from doing so outweighs the health risks. Thus, in this instance, smoking can be viewed as a utility-maximising behaviour ceteris paribus. However, we also know that not everyone in such a friend group will take up smoking. Why not? In norm-dependent utility theories, an individual’s perceived utility of smoking with friends is scaled by the individual’s sensitivity to norms. If the adolescent is highly sensitive to the prevailing norms of the friendship group, he/she will conform to the prescribed behaviours deemed to yield the greatest level of utility, thereby opting to smoke. Conversely, if he/she is not very sensitive to the norm, his/her choices may not be affected by the desire to avoid the utility cost of violating the norm, and therefore he/she does not choose to smoke. By analogy, this carries over to various risk behaviours. Furthermore, by allowing for individual differences in norm-following proclivity, this example belies the notion that exposure to risk behaviour directly predicts participation. Consequently, the decision is now characterised by a set of utility functions differentiated by a single parameter that captures individuals’ heterogeneous disutility from violating the norms.

It is well documented that adolescents are increasingly exposed to similar risk-taking during this period of development^11^, particularly in the presence of peers^12^. However, as we have noted, a limited number of studies have attempted to argue for the role of sensitivity to norms in determining behavioural choices. Even less is known about the personality and cognitive traits that govern this sensitivity. Research has shown that adolescents consistently exhibit disproportionately higher levels of reward-seeking^13^ and risk-taking behaviours^14,15^. Moreover, risk-taking preferences can be shaped by sensitivity to reward^16,17^. Somerville et al.^18^ posit that “risky behaviors observed in adolescence are likely related to an enhanced motivation to seek out incentives and new experiences” (p. 125). Hence it is not untenable to suggest that incentive-driven behaviour and sensitivity to norms share similar underlying causal properties.

A study by Altikulaç et al.^19^ found that sensitivity to social reward was determined by a complex interaction between age and gender, with older adolescents being driven by a desire to be liked and to gain positive attention from others. Other studies have identified male adolescents as being more susceptible to peer influence than their female counterparts^20,21^. This is in line with the findings of Meldrum et al.^22^ who additionally observed that susceptibility to peer influence was negatively associated with self-control. Sumter et al.^23^ reported in their study that girls displayed a greater resistance to peer influence while a general increase in resistance to peer influence was recorded among both male and female participants. Kimbrough and Vostroknutov found that females had higher levels of norms sensitivity in rule-following tasks^24,25^. An earlier study by Raffaelli et al.^26^ found that girls possessed higher self-regulatory ability than boys, however, the authors note that the factors underpinning this gender difference are not known. This sentiment was echoed by Allen et al.^27^ who concluded: “…susceptibility may reflect underlying developmental difficulties that produce both susceptibility and problematic outcomes… Further research is now needed to understand the sources of such susceptibility and its exact relation to future problematic behaviors” (p. 169).

Despite studies demonstrating variability in adolescents’ susceptibility to influence^28^, the determinants and gender-related differences of this susceptibility are less clear. In addition, norm-following propensity is not a well understood construct, nor has it been sufficiently researched in the context of public health. Conversely, social norms are investigated with relative consistency as a predictor of multiple risk behaviours that include smoking^29,30^; drug use^31–33^; and alcohol use^34,35^. Nonetheless, few studies have harnessed novel empirical tools such as those adopted in this study to elicit individual sensitivity to norms with the aim of understanding which personality and cognitive traits predispose adolescents to the influence of social norms. We set out to address this gap in the literature through the use of a modified version of the Rule-Following (RF) task validated by Kimbrough and Vostroknutov^24^. The following research questions were addressed:

- H_1_: Is adolescents’ sensitivity to norms associated with various personality and cognitive traits?
- H_2_: Does the distribution and magnitude of these associations vary across male and female participants?
- H_3_: After controlling for personality and cognitive traits, does gender retain a significant association with sensitivity to norms?

## Methods

### Study population

Study participants were a cross-sectional sample from the first wave of data collection of the Mechanisms of Networks and Norms Influence on Smoking in Schools (MECHANISMS) study. The MECHANISMS study was a school-based study designed to further understanding of social norms based mechanisms of action related to smoking in high- and middle-income settings. Two waves of data collection took place in Northern Ireland and Bogotá (Colombia) before and after students participated in school-based smoking prevention interventions (see Hunter et al.^36^ for full details of the study design).

Cross-sectional data were collected from 1274 students aged 11–15 years in a post-primary educational setting taking part in a structured smoking prevention programme in schools in Northern Ireland, UK (n = 6) and Bogotá (n = 6). In Northern Ireland, the sample of schools served urban and rural catchments, and maximum variation sampling was used to ensure there was an adequate balance of schools with high and low proportions of pupils eligible for free school meals. Eight public schools in Bogotá were identified using a comparable maximum variation sampling approach. Sampling of schools in Bogotá was performed in three steps: first, 40 private and public schools were identified based on health risks by the Education and Health Departments of Bogotá; second, 13 schools were shortlisted for inclusion in the study if they were situated in an urban area, were mixed-gender, and had an enrolment of 90–150 students in year 7; third, six schools accepted the invitation to participate in the study and were subsequently selected. All data were collected on portable tablets using Qualtrics. Survey instruments were previously translated and adapted to Spanish-speaking populations (see Part A and B of the “Appendix” for further information on study procedures and data collection).

### Assessment of sensitivity to norms

General norms sensitivity was measured using a variant of the incentivised Rule-Following task presented by Kimbrough and Vostroknutov^24,25^ designed to assess participants’ propensity to follow established rules and social norms. Monetary incentives were adjusted for purchasing power parity, aim of the game, and instructions compressibility in Bogotá. All payments were delivered in cash in Northern Ireland and in a gift card in Bogotá at the conclusion of the MECHANISMS study.

Participants were presented with a task of allocating 50 balls across two buckets (one blue and one yellow) and instructed that “The rule is to put the balls in the blue bucket”. Participants could choose freely how to allocate the share of balls to each bucket within the stipulated time frame of five minutes, with no risk of penalty for violating the stated rule.

The specific monetary value attached to each bucket was as follows: each ball placed in the blue bucket was worth 5p in Northern Ireland and 100 Colombian Pesos (COP) in Bogotá; and each ball placed in the yellow bucket was worth 10p in Northern Ireland and 200 COP in Bogotá. Unallocated balls were worth nothing. The maximum amount a participant could earn if he/she followed the rule completely was £2.50 in Northern Ireland and 5000 COP in Bogotá compared to £5.00 and 10,000 COP in the two countries respectively if they ignored the stated rule completely. Therefore, by following the rule, participants incurred an explicit monetary cost proportional to the degree of rule-following (for an example of the survey instrument see Part C of the “Appendix”).

This task provides an incentivized measure of participants’ willingness to incur a monetary cost in order to follow an arbitrary, experimenter-stated rule. If individuals prefer more money to less and if there is a normative expectation that participants in a study ought to comply with the requests of a researcher, then the extent of rule-following in the task proxies for an individual’s propensity to follow injunctive norms. Indeed, behaviour in the RF task has been shown to predict norm-consistent behavior across a variety of tasks (see Kimbrough and Vostroknutov 2015^37^, 2016^24^, 2018^25^; Ridinger 2018^38^; Gross and DeDreu 2021^39^).

The extent of rule-following in the RF task provides a measure of individual norm-following proclivity, and this norm sensitivity measure has been shown to correlate with willingness to follow norms of cooperation, reciprocity and prosocial behaviour across decision contexts. To avoid introducing any potential biases due to preference for bucket placement, participants were randomized to a version of the RF task with the blue bucket on the left (n = 621), or a version with the blue bucket on the right (n = 650).

The distribution of the dependent variable did not follow a normal distribution (see Part D of the “Appendix”) and in line with Kimbrough and Vostroknutov^25^ we classified “full rule-following” individuals as those who allocated 50 balls to the blue bucket and “full rule-breaking” individuals as those who allocated 0 balls to the blue bucket. Our approach deviates from that of Kimbrough and Vostroknutov with the inclusion of three new levels, transforming the dependent variable into an ordinal variable with the adolescents being organised into five sensitivity to norms levels based on their scores in the rule-following task (see Table 1).

**Table 1:** Distribution of sensitivity to norms levels.

	Rule-following	Total	Male	Female
1	Full rule-breaking	151 (12.4%)	110 (18.4%)	36(6.0%)
2	Prefer rule-breaking	217 (17.9%)	118 (19.7%)	99 (16.5%)
3	Neutral	169 (13.9%)	82 (13.7%)	85 (14.1%)
4	Prefer rule-following	296 (24.3%)	106 (17.7%)	187 (31.1%)
5	Full rule-following	383 (31.5%)	183 (30.6%)	195 (32.4%)

Sensitivity to norms was originally designed to be measured on a continuous scale (0–50), and linear mixed effects models were included as a supplementary analyses of the associations between the personality/cognitive traits and sensitivity to norms (see Part G of the “Appendix”).

### Assessment of personality and cognitive traits

We assessed the “Big Five” adolescent personality traits^40^ by using the Big Five Personality Trait Short Questionnaire (BFPTSQ). Students were asked to express their agreement with a list of statements pertaining to different domains of personality by selecting options on a five-point Likert scale. Each dimension was measured using a 10-item subscale: openness (Cronbach’s α = 0.798); extraversion (Cronbach’s α = 0.776); agreeableness (α = 0.700); conscientiousness (α = 0.700); and emotional stability (Cronbach’s α = 0.745). In Northern Ireland, we used the questionnaire validated for English-speaking adolescents^41^. In Bogotá, we used the questionnaire validated for Spanish-speaking adults^42^.

The Prosociality score was derived from 5 items from the self-report version of the Strengths and Difficulties Questionnaire (SDQ) (e.g., “I try to be nice to other people. I care about their feelings”) measured on a three-point scale from 0 = “not true” to 2 = “certainly true”, with a higher score reflecting more prosocial behavioural preferences^43,44^ (Cronbach’s α = 0.733).

The Need to Belong score was derived from the 10-item Need to Belong Scale (NTBS) (e.g., “If other people don't seem to accept me, I don't let it bother me”) measured on a five-point scale, with a higher score indicating a greater need to belong^44,45^ (Cronbach’s α = 0.813).

The Fear of Negative Evaluation score was derived from the 12-item brief version of the Fear of Negative Evaluation Scale (e.g., “I am afraid others will not approve of me”) measured on a five-point scale, with a higher score indicating a greater fear of negative evaluation^44,46,47^ (Cronbach’s α = 0.894).

### Assessment of sociodemographic characteristics

Sociodemographic data collected at wave one included country, gender (students who chose the option “prefer not to say” were coded as missing), age, ethnicity, and family structure.

### Statistical analysis

As the first stage of statistical analysis, descriptive statistics were used to analyse frequency, percentage, and mean values for sensitivity to norms, and personality and cognitive traits. After checking homogeneity of variance using Levene’s test, a series of independent samples t-tests and Welch’s t-tests were performed to assess if there was a statistically significant difference in mean scores for each of the personality and cognitive traits across genders. A chi-squared test was used to determine if sensitivity to norms level differed significantly across the two subgroups.

One way analysis of variance (ANOVA) tests ( “Appendix” Part E) were carried out to determine whether there was a significant difference between mean scores for the personality and cognitive traits across each sensitivity to norms level.

After checking distributional assumptions, a series of multilevel mixed effects ordered logistic regression models were computed (see Part F of the “Appendix”). However, given that the norm-dependent utility function used in this experiment was operationalised as a continuous measure of each individual’s rule-following proclivity, multilevel linear mixed effects models were computed as supplementary analyses (see Part G of the “Appendix”). By utilising both linear and ordered logistic models, we allow for: (1) the way in which sensitivity to norms was originally intended to be used; and (2) the distributional assumptions for linear models being violated.

Multilevel mixed effects ordered logistic regression models were used to examine the association between the personality and cognitive traits of adolescents and their sensitivity to norms level. Given that the data were nested, a 4-level hierarchical model was used. This involved students (level 1, n = 1258) nested in classes (level 2, n = 55), classes nested in schools (level 3, n = 12), and schools nested in countries (level 4, n = 2). The multilevel models were computed with three random intercepts at Country-, School-, and Class-level. The independent variables were computed as fixed effects in each multilevel model. Using one-way analysis of variance, the intraclass correlation coefficient (ICC) for between-Country difference was 0.01503 in sensitivity to norms scores. ICCs computed for Schools (0.03334) and Classes (0.12655) were similarly low, suggesting that there was low levels of clustering within schools and classes.

A three-step multilevel mixed-effects ordered logistic regression was computed using sensitivity to norms level as the dependent variable. In the first step, univariate models assessed the relationship between the independent variables (openness; extraversion; agreeableness; conscientiousness; emotional stability; need to belong; fear of negative evaluation; prosociality; and gender) computed as fixed effects and sensitivity to norms level with random intercepts at Country-, School- and Class-level. In the second step, a multivariate model was computed by simultaneously entering all the independent variables as fixed effects while maintaining random intercepts at Country-, School- and Class-level. The third model introduced interaction terms to determine if there was a statistically significant difference in predictors of sensitivity to norms level according to gender. Following this, the data were stratified by gender, and the same set of analyses carried out for both male and female participants.

For the multivariate regression models, raw independent variables were converted to z-scores to accommodate differences in instrument scales.

All statistical analyses were conducted using Stata 16.1 (StataCorp, 2019, Stata Statistical Software: Release 16. College Station, TX: StataCorp LLC.).

### Ethics approval and consent to participate

The studies involving human participants were reviewed and approved. Ethical approval was granted from the Queen's University Belfast School of Medicine, Dentistry and Biomedical Sciences Ethics Committee (reference number 18.43; v3 Sept 21, 2018), and Research committee of the Universidad de Los Andes, Bogotá (937;July 30, 2018). Written informed consent to participate in this study was provided by the participants' legal guardian/next of kin.

The methods were carried out in accordance with the relevant guidelines and regulations set out in the Declaration of Helsinki.

## Results

Descriptive statistics are shown in Table 2. The mean score in the rule-following task was 31.00 for the sample overall, while females scored higher on average than males (33.85 vs 28.23 respectively). A Welch’s t-test test confirmed that there was a statistically significant difference in sensitivity to norms according to gender (p < 0.000). In addition, a larger proportion of males opted to disregard the rule of the game completely (18%) compared to females (6%). Another noteworthy difference was found in the proportion of females who were classified as “prefer rule-following” (31%) compared to males (18%). A Chi-squared (χ^2^) test (p < 0.001) of the categorical data supported the results of the Welch’s t-test indicating that sensitivity to norms different significantly across the two subgroups.

**Table 2:** Descriptive statistics.

Sample characteristics^a^	Overall (n = 1258)		Males (n = 625)		Females (n = 633)		T-test p-value
**Demographic characteristics**							
Age, years^b^							0.005^c^
11	7	(1%)	1	(0%)	6	(1%)	
12	459	(36%)	213	(34%)	240	(38%)	
13	632	(50%)	303	(48%)	319	(50%)	
14	112	(9%)	68	(11%)	44	(7%)	
15 or more	64	(5%)	40	(6%)	24	(4%)	
Ethnicity^b^							0.671^c^
Non-ethnic minority	1137	(89%)	556	(89%)	566	(90%)	
Ethnic minority	135	(11%)	69	(11%)	65	(10%)	
Household composition^b^							0.649^c^
Non-Single parent	908	(71%)	442	(71%)	455	(72%)	
Single parent	366	(29%)	183	(29%)	178	(28%)	
**Sensitivity to norms**							
Blue bucket allocation	31.00	(18.0)	28.23	(19.2)	33.85	(16.1)	< 0.001^d^
Full rule-breaking^b^	151	(12%)	110	(18%)	36	(6%)	< 0.001^c^
Prefer rule-breaking^b^	217	(18%)	118	(20%)	99	(16%)	
Neutral^b^	169	(14%)	82	(14%)	85	(14%)	
Prefer rule-following^b^	296	(24%)	106	(18%)	187	(31%)	
Full rule-following^b^	383	(32%)	183	(31%)	195	(32%)	
**Big five personality traits**							
Openness	2.57	(0.69)	2.49	(0.72)	2.64	(0.65)	< 0.001^d^
Extraversion	2.63	(0.74)	2.62	(0.73)	2.65	(0.76)	0.538
Agreeableness	2.58	(0.65)	2.51	(0.64)	2.64	(0.65)	0.001
Conscientiousness	2.34	(0.66)	2.32	(0.65)	2.36	(0.66)	0.222
Emotional stability	1.99	(0.76)	2.16	(0.71)	1.83	(0.77)	< 0.001
**Other cognitive traits**							
Prosociality	7.71	(2.13)	7.34	(2.25)	8.08	(1.95)	< 0.001^d^
Need to belong	2.95	(0.63)	2.90	(0.61)	3.01	(0.64)	0.004
Fear of negative evaluation	2.77	(0.65)	2.70	(0.58)	2.84	(0.70)	< 0.001^d^

^a^Variable distributions are reported as mean (standard deviation) unless otherwise stated.

^b^Variable distributions are reported as n (%).

^c^Chi-squared (χ^2^) test.

^d^Welch’s t-test.

Of the Big Five personality traits, t-tests identified significant differences across the male and female subgroups for openness, agreeableness, and emotional stability. On average, females scored higher than males on both openness and agreeableness scales. Conversely, males scored higher than females on the emotional stability scale. No statistically significant differences were observed for extraversion and conscientiousness between the two subgroups.

Differences were also observed for other cognitive traits, with females scoring higher than males on average on the prosociality, need to belong, and fear of negative evaluation scales.

### H_1_: Is adolescents’ sensitivity to norms associated with various personality and cognitive traits?

The one-way analysis of variance results (shown in Table S1, “Appendix” Part E) showed significant differences across sensitivity to norms levels for openness, agreeableness, and fear of negative evaluation. This finding is further reinforced when examining the mean scores for the three traits at each sensitivity to norms level (Table S2, “Appendix” Part E). Adolescents who were classified as full rule-breaking (i.e., score = 0 in the rule-following task) had the lowest mean score on the openness scale (2.44) compared with those who were neutral (2.56) and full rule-followers (2.63). This trend was similar for agreeableness, with adolescents who were classified as full rule-breaking exhibiting the lowest mean score on the agreeableness scale (2.44) when compared to those who were neutral (2.65) and full rule-followers (2.63). By contrast, fear of negative evaluation demonstrated an inverse relationship with the ordinal scale for sensitivity to norms. Adolescents who were classified as full rule-breaking had the highest mean score on the fear of negative evaluation scale (2.87), whereas adolescents who were classified as neutral and full rule-following had lower mean scores (2.84 and 2.73 respectively).

The results of the univariate multilevel mixed effects ordered logistic regression analyses are shown in Table S5 ( “Appendix” Part F). Of the Big Five personality traits, higher scores for openness (β = 0.19, z = 2.33, p = 0.020) and extraversion (β = 0.18, z = 2.31, p = 0.021) significantly increased the log odds of being in a higher sensitivity to norms level. None of the cognitive traits were found to have a statistically significant association with sensitivity to norms in the univariate multilevel mixed effects ordered logistic regression models.

The results of the multivariate multilevel mixed effects ordered logistic regression model are shown in Table S6 ( “Appendix” Part F). In the overall sample, none of the personality or cognitive traits demonstrated a statistically significant association with sensitivity to norms level.

### H_2_: Does the distribution and magnitude of these associations vary across male and female participants?

When the results of the ANOVA tests were stratified by gender (Table S3 and Table S4, “Appendix” Part E), they were largely similar to that of the composite sample except for a few noteworthy deviations: for the male sample, ANOVA results indicated there was no significant difference between each sensitivity to norms level for scores on the openness scale; and in the female sample, the only significant difference was found for fear of negative evaluation among the five sensitivity to norms levels.

Interaction analyses of the multilevel mixed effects ordered logistic regression models (Tables S7 and S8, “Appendix” Part F) showed significant univariate interactions with gender (p ≤ 0.05) for conscientiousness, emotional stability, and fear of negative evaluation. In the multivariate model, significant interactions with gender were present for emotional stability, need to belong and fear of negative evaluation. These results illustrate that, particularly in the multivariate ordered logistic regression model, the association between sensitivity to norms and traits such as emotional stability, need to belong and fear of negative evaluation may be moderated by gender.

Results of the gender-stratified univariate multilevel mixed effects ordered logistic regression models (Table S5, “Appendix” Part F) indicated that, in the male subgroup, the Big Five personality traits that demonstrated statistically significant positive log odds were agreeableness (β = 0.28, z = 2.19, p = 0.028) and conscientiousness (β = 0.30, z = 2.36, p = 0.018). Fear of negative evaluation (β = -0.51, z = − 3.41, p = 0.001) was the only cognitive trait among males that significantly negatively predicted the log odds of sensitivity to norms level. In the female subgroup, no independent variables showed a significant association with sensitivity to norms level in the univariate ordinal models.

The results of the gender-stratified multivariate multilevel mixed effects ordered logistic regression analyses (Table S6, “Appendix” Part F) showed that need to belong (β = -0.21, z = − 1.97, p = 0.049) negatively predicted the log odds of being in a higher sensitivity to norms level among males. For female respondents, the multivariate model indicated that emotionally stability (β = − 0.21, z = − 2.11, p = 0.035) was significantly negatively associated with sensitivity to norms level.

### H_3_: After controlling for personality and cognitive traits, does gender retain a significant association with sensitivity to norms?

In the univariate multilevel mixed effects ordered logistic regression model, being female significantly increased the log odds of being classified in a higher rule-following level (β = 0.46, z = 4.22, p < 0.001).

After adjusting for all other independent variables, gender (β = 0.42, z = 3.48, p = 0.001) significantly predicted the log odds of sensitivity to norms level, with females being more likely to be categorised in a higher rule-following level compared to males.

## Discussion

The goal of this study was to understand if adolescents’ sensitivity to social norms was associated with a variety of personality and cognitive traits, and whether these associations varied according to gender. Overall, female adolescents had higher levels of sensitivity to norms. With the exception of gender as a predictor of sensitivity to norms, the results showed no consistent pattern between sensitivity to norms and the personality and cognitive traits. Results of the gender-stratified analyses indicated that need to belong was negatively associated with sensitivity to norms in males, while emotional stability was negatively associated with sensitivity to norms for females. This was supported by interaction analyses that showed significant interactions for emotional stability, need to belong and gender, further highlighting the potential moderating role of gender between some psychosocial traits and sensitivity to norms.

### Gender differences in sensitivity to norms and other traits

In the present study there were a number of significant mean-level differences between males and females with regards to the various personality and cognitive traits. Females scored significantly higher than males on both prosociality and agreeableness scales, in line with results from earlier studies^48–54^. However, contrary to the findings of Uliaszek et al.^55^ and Klimstra et al.^56^ females and males had similar extraversion scores, and mean levels of conscientiousness were comparable, counter to results from Iimura and Taku^57^.

Similar to other studies, the males in the sample reported higher levels of emotional stability^48,52^. On the other hand, self-reported fear of negative evaluation was higher among female adolescents, corroborating the results of other studies^58–60^ and which may be linked to higher levels of social anxiety in adolescent girls^61,62^; lower self-esteem^63^; or higher sensitivity to rejection^64^. Openness and need to belong were additional traits on which females had higher scores. As such, the findings align with those of Shi et al.^65^ and Leibovich et al.^66^, respectively.

Consistent with Kimbrough and Vostroknutov^24,25^ gender was the only factor that demonstrated a significant association with norms sensitivity in the multivariate multilevel mixed effects ordered logistic regression model. Further, the results point to a moderating role of gender between traits such as emotional stability and need to belong and sensitivity to norms, suggesting that gender may strengthen or attenuate the relationship between some traits and sensitivity to environmental normative influence.

These gender differences are, in part, explained in a review by McCoy et al.^67^ who draw on gender role socialization theory to explain the differences in how gender norms and expectations are socially conditioned and configured around gender-specific stereotypes. According to this view, males may be more susceptible to deviant behaviour during adolescence when gender role stereotypes become more salient, and rule-breaking behaviours are viewed as a means of garnering approval among male peers^68,69^. Contrastingly, characteristics associated with female stereotypes such as nurturance and emotional lability, in combination with greater social sanctions for deviant behaviour, place them at reduced risk of deviant behaviour^70^. This is supported by identity theorists who contend that parental expectations foster certain personality traits (or identity standards) such as prosociality and conscientiousness in female children, whereas male children are expected to adopt more assertive behavioural characteristics in pursuit of greater autonomy and self-reliance^71^.

As a potential moderator of the relationship between external influences and intentions to engage in risky behaviour, sensitivity to norms may contribute to healthy development if it enables adolescents to regulate social behaviours and make sound judgements about the potential consequences, and thus can be linked to goal-oriented behavioural preferences for adolescents. For instance, compared to males, adolescent females are more prosocial and exhibit more “connection-oriented” (communal) goals, valuing relationships with others and demonstrating greater interpersonal sensitivity^72^. Consequently, the rule-following task employed in this study may have elicited those values among the female participants. By comparison, male adolescents are largely driven by agentic goals characterised by a desire for leadership and status^73^.

These assumptions bifurcate development into two broad dimensions of agency and communion^74^. As proposed by gender intensification theory^75^, gender role identities and behaviours diverge during early adolescence as a result of social pressure to conform to socially sanctioned gender roles. Therefore, male adolescents more commonly identify themselves with agentic attributes such as strength and intelligence, while female adolescents identify with communal traits such as being helpful, sensitive, and communicative^76^.

### Adolescent choice preferences and health behaviour

While sensitivity to norms and susceptibility to peer influence are not the same, the findings reveal important differences in how approaches to the rule-following task differed across the two genders. Furthermore, the absence of any statistically significant associations between sensitivity to norms and the personality and cognitive traits implies that sensitivity to norms may be an independent, and stable, personality dimension. Equally as pertinent is the observation that, proportionately speaking, the same number of males and females opted to completely follow the rule in their respective subgroups. Only among the males was there a significantly larger proportion choosing to ignore the rule completely. Therefore, three possibilities emerge: (a) the disutility from violating the stated rule of the game in exchange for greater monetary reward was regarded as acceptable among males, suggesting there was an intrinsic motivation for personal gain outweighing any disutility of violating the rule (and its concomitant normative expectation); (b) females experienced greater disutility at the prospect of violating the rule, irrespective of the monetary consequences; or (c) some combination of (a) and (b).

Models of norm-dependent utility assume that the normative valence of an outcome is captured in a single index of “social appropriateness” which enters into decision-making, weighted by individual norm-sensitivity. But such a model leaves the sources of “social appropriateness” inside a black box. One factor worth considering is how social appropriateness depends on an individual’s reference group and how different groups of which an individual sees him/herself as a member may make competing normative demands on behaviour—e.g., the norms of one’s friend group may differ from the norms of one’s family such that following friends’ norms means violating their family’s. Explicit consideration of these factors may help explain “audience effects”, or why finding an unopened pack of cigarettes is more likely to result in smoking uptake when amongst one’s peers than when with one’s family. Moreover, such models might be fruitfully combined with work on social reward processing from the field of social neuroscience, and further complemented by robust measures of how the perceived social value of performing an action (and other salient sociocontextual factors) can alter subjective assessments of utility and risk. For example, reward processing can be influenced by the presence of others^77^ or by the context within which decisions are framed^78^. Ciranka and van den Bos^79^ adapted the expected utility model to three processes underpinning social influence: (1) social motivation; (2) reward sensitivity; and (3) distraction. Each modification to the base utility function adds parameters that can account for the three different social influence processes. Specifically, the social sensitivity and reward sensitivity parameters make allowances for social information and social context—two considerations that are important during adolescence.

### Implications for future research

One implication of the norm-dependent preferences framework is that people will do whatever is seen as normative in their reference group. Depending on the reference group, some behaviours can be viewed as more deviant than others. For example, antisocial peer behaviours (e.g., vandalism) carry different implications than prosocial peer behaviours (e.g., donating to charity). With this in mind, we recall an earlier experimental study by Van Hoorn et al.^80^ that employed a public goods game to examine how prosocial behaviours of adolescents were affected by peer influence. The results indicated that peers could encourage both prosocial and antisocial behavioural choices. Therefore, future research should consider the application of similar choice-based models while integrating tasks that are more aligned with a variety of conventional risk behaviours. That is not to suggest that adolescents’ risk-taking preferences are directly linked to sensitivity to norms. Rather, the results of the current study provide a basis for further empirical research on a relatively nascent construct, and so far as extant research surrounding adolescent decision-making is concerned, bring a fresh perspective to the question of norm-following preferences among this age group, albeit with a focus on a limited number of personality and cognitive traits.

Given the well documented differences in risk-taking between males and females^81^, there is still an open question as to what extent this is related to rule-following. The results from this study suggest that when risky behaviors become normative, females (not males) would be more prone to engage in them, since they are more sensitive to norms. Males may have different intrinsic attitudes towards risk, which lead them to take more risk whether doing so is normative or not, but the model of norm-dependent utility assumes that risk preferences and norm-sensitivity are orthogonal (as long as the latter is not measured under conditions where there is a threat of punishment, which would confound norm-sensitivity with risk).

The field of social neuroscience has provided many innovative and novel contributions to the scientific discourse around adolescent health behaviour. This is exemplified in many studies’ application of functional and structural magnetic resonance imaging (MRI) to capture complex neurobiological processes that underpin susceptibility to social influence during adolescence (see Telzer et al.^82^). For instance, Steinberg’s^83^ research addresses the question of why risk-taking increases in the years leading to adolescence and subsequently declines prior to adulthood. Steinberg submits that alterations in the brain’s socio-emotional system during puberty heightens adolescents’ sensitivity to reward-related stimuli. This is brought about by a reconditioning of the brain’s dopaminergic system, increasing the vulnerability of adolescents to contextual influence^84^. This is echoed by other researchers who also confirm a pattern of heightened sensitivity during adolescence to social rewards^85,86^. Important gender differences have also been identified in behavioural neuroscience research. For example, Alarcón et al.^87^ showed that during a series of self-referential processing tasks, neural responses among girls indicated higher levels of disruption between neural networks that support self-referential processing and cognitive control.

Another notable result was that need to belong was significantly associated with sensitivity to norms for males. By comparison, emotional stability was significantly associated with sensitivity to norms for females. This adds weight to our findings in the interaction analyses, which suggested that the relationship between these traits and sensitivity to norms is potentially moderated by gender. Further research should attempt to uncover the potential mechanistic pathways between these traits and sensitivity to norms with a formal mediation/moderation analysis.

### Strengths and limitations

There were a number of strengths associated with this study. First, the study used an independent task to elicit sensitivity to norms, so the data are unconfounded with contextual cues present in many games that might influence measured sensitivity^24^. Second, the study drew from a large sample of adolescent students from two settings with varying sociocultural characteristics and health behaviour patterns.

This study also has several limitations. The data are cross-sectional, therefore any causal inferences could not be made. There were also several caveats associated with using a single task to assess sensitivity to norms. Social norms are complex, and their influence varies significantly according to behaviour and context. As such, future research should incorporate a range of experimental tasks that capture sensitivity to different types of norms such as injunctive norms, subjective norms, or descriptive norms. This may create a more robust measure of norms sensitivity.

As noted earlier, sensitivity to norms was originally conceived to be measured on a continuous scale. Thus, we are reticent to rely solely on the findings of the ordinal model alone as transforming sensitivity to norms into an ordinal variable collapses the data and discards useful information provided by the continuous scale version. Further, we contend that any divergence in the results between the linear models and ordinal models might suggest scope for further research or refinement of the experimental tool to capture norms sensitivity. Therefore, we suggest that inconsistencies across the linear and ordinal models do not preclude the ability to draw statistically meaningful conclusions from the data.

## Conclusions

The purpose of this study was to understand if various personality and cognitive traits were associated with sensitivity to social norms, and whether these associations were different according to gender. The results reinforced the findings from an earlier study and suggested female adolescents had higher levels of sensitivity to norms. With the exception of gender, the results showed no significant associations between sensitivity to norms and any of the personality and cognitive traits. However, the results provide a basis for future studies to examine the psychosocial determinants of this relatively novel construct. Future research in this area would benefit from examining norm-following preferences as they correspond to different health behaviours. Moreover, there are opportunities for greater interdisciplinary consilience in this area. For example, advances being made in social neuroscience to understand more about social reward processing can complement the norm-dependent utility models advocated in this study.

## Supplementary Information

Supplementary Information.

## Data Availability

The datasets used and/or analysed during the current study are available from the corresponding author on reasonable request.

## References

[1] Berndt, T. J. Transitions in friendship and friends’ influence. In *Transitions Through Adolescence: Interpersonal Domains and Context.* (eds Graber, J. A., Brooks-Gunn, J. & Petersen, A. C.) 57–84 (Lawrence Erlbaum Associates, Inc, 1996).

[2] Blakemore S-J, Mills KL (2014). Is adolescence a sensitive period for sociocultural processing?. Annu. Rev. Psychol..

[3] Hansell, S. & Mechanic, D. 3. Parent and peer effects on adolescent health behavior. In *Health Hazards in Adolescence* (eds Lösel, F. & Hurrelmann, K.) 43–66 (De Gruyter, 1990). 10.1515/9783110847659-004.

[4] Cialdini RB, Kallgren CA, Reno RR (1991). A focus theory of normative conduct: A theoretical refinement and reevaluation of the role of norms in human behavior. Adv. Exp. Soc. Psychol..

[5] Blakemore S-J (2018). Avoiding social risk in adolescence. Curr. Dir. Psychol. Sci..

[6] Nelson EE, Jarcho JM, Guyer AE (2016). Social re-orientation and brain development: An expanded and updated view. Dev. Cogn. Neurosci..

[7] Brechwald WA, Prinstein MJ (2011). Beyond homophily: A decade of advances in understanding peer influence processes. J. Res. Adolesc..

[8] Prinstein MJ, Brechwald WA, Cohen GL (2011). Susceptibility to peer influence: Using a performance-based measure to identify adolescent males at heightened risk for deviant peer socialization. Dev. Psychol..

[9] Teunissen HA (2016). Friends’ drinking norms and male adolescents’ alcohol consumption: The moderating role of performance-based peer influence susceptibility. J. Adolesc..

[10] Do, K. T., Prinstein, M. J. & Telzer, E. H. Neurobiological susceptibility to peer influence in adolescence. In *The Oxford Handbook of Developmental Cognitive Neuroscience* (ed Cohen Kadosh, K.) (Oxford University Press, 2020). 10.1093/oxfordhb/9780198827474.013.27.

[11] Lightfoot C (1997). The Culture of Adolescent Risk Taking.

[12] Gardner M, Steinberg L (2005). Peer influence on risk taking, risk preference, and risky decision making in adolescence and adulthood: An experimental study. Dev. Psychol..

[13] Steinberg L (2010). A dual systems model of adolescent risk-taking. Dev. Psychobiol..

[14] Eaton D (2008). Youth risk behavior surveillance—United States, 2007. MMWR. Surveill. Summ..

[15] Figner B, Mackinlay RJ, Wilkening F, Weber EU (2009). Affective and deliberative processes in risky choice: Age differences in risk taking in the Columbia Card Task. J. Exp. Psychol. Learn. Mem. Cogn..

[16] Reniers RLEP, Murphy L, Lin A, Bartolomé SP, Wood SJ (2016). Risk perception and risk-taking behaviour during adolescence: The influence of personality and gender. PLoS ONE.

[17] Freeman C, Dirks M, Weinberg A (2020). Neural response to rewards predicts risk-taking in late but not early adolescent females. Dev. Cogn. Neurosci..

[18] Somerville LH, Jones RM, Casey BJ (2010). A time of change: Behavioral and neural correlates of adolescent sensitivity to appetitive and aversive environmental cues. Brain Cogn..

[19] Altikulaç S, Bos MGN, Foulkes L, Crone EA, van-Hoorn J (2019). Age and gender effects in sensitivity to social rewards in adolescents and young adults. Front. Behav. Neurosci..

[20] Mears DP, Ploeger M, Warr M (1998). Explaining the gender gap in delinquency: Peer influence and moral evaluations of behavior. J. Res. Crime Delinq..

[21] Widman L, Choukas-Bradley S, Helms SW, Prinstein MJ (2016). Adolescent susceptibility to peer influence in sexual situations. J. Adolesc. Heal..

[22] Meldrum RC, Miller HV, Flexon JL (2013). Susceptibility to peer influence, self-control, and delinquency. Sociol. Inq..

[23] Sumter SR, Bokhorst CL, Steinberg L, Westenberg PM (2009). The developmental pattern of resistance to peer influence in adolescence: Will the teenager ever be able to resist?. J. Adolesc..

[24] Kimbrough EO, Vostroknutov A (2016). Norms make preferences social. J. Eur. Econ. Assoc..

[25] Kimbrough EO, Vostroknutov A (2018). A portable method of eliciting respect for social norms. Econ. Lett..

[26] Raffaelli M, Crockett LJ, Shen Y-L (2005). Developmental stability and change in self-regulation from childhood to adolescence. J. Genet. Psychol..

[27] Allen JP, Porter MR, McFarland FC (2006). Leaders and followers in adolescent close friendships: Susceptibility to peer influence as a predictor of risky behavior, friendship instability, and depression. Dev. Psychopathol..

[28] Telzer EH, Jorgensen NA, Prinstein MJ, Lindquist KA (2021). Neurobiological sensitivity to social rewards and punishments moderates link between peer norms and adolescent risk taking. Child. Dev..

[29] Xuefen S (2015). Smoking behaviors and intentions among adolescents in rural China: The application of the Theory of Planned Behavior and the role of social influence. Addict. Behav..

[30] Lozano P, Arillo-Santillán E, Barrientos-Gutíerrez I, Reynales-Shigematsu LM, Thrasher JF (2019). E-cigarette social norms and risk perceptions among susceptible adolescents in a country that bans E-cigarettes. Heal. Educ. Behav..

[31] Hohman ZP, Crano WD, Siegel JT, Alvaro EM (2014). Attitude ambivalence, friend norms, and adolescent drug use. Prev. Sci..

[32] Roditis ML, Delucchi K, Chang A, Halpern-Felsher B (2016). Perceptions of social norms and exposure to pro-marijuana messages are associated with adolescent marijuana use. Prev. Med. (Baltim).

[33] Barnum TC, Armstrong T (2019). Sensation seeking to marijuana use: Exploring the mediating roles of risk appraisal and social norms. Addict. Behav..

[34] Litt DM, Stock ML (2011). Adolescent alcohol-related risk cognitions: The roles of social norms and social networking sites. Psychol. Addict. Behav..

[35] Mahalik JR, Lombardi CM, Sims J, Coley RL, Lynch AD (2015). Gender, male-typicality, and social norms predicting adolescent alcohol intoxication and marijuana use. Soc. Sci. Med..

[36] Hunter RF (2020). MECHANISMS study: Using game theory to assess the effects of social norms and social networks on adolescent smoking in schools—study protocol. Front. Public Heal..

[37] Kimbrough EO, Vostroknutov A (2015). The social and ecological determinants of common pool resource sustainability. J. Environ. Econ. Manage..

[38] Ridinger G (2018). Ownership, punishment, and norms in a real-effort bargaining experiment. J. Econ. Behav. Organ..

[39] Gross J, De Dreu CKW (2021). Rule following mitigates collaborative cheating and facilitates the spreading of honesty within groups. Personal. Soc. Psychol. Bull..

[40] Goldberg LR (1990). An alternative ‘description of personality’: The big-five factor structure. J. Pers. Soc. Psychol..

[41] Morizot J (2014). Construct validity of adolescents’ self-reported big five personality traits: Importance of conceptual breadth and initial validation of a short measure. Assessment.

[42] Ortet G, Martínez T, Mezquita L, Morizot J, Ibáñez MI (2017). Big five personality trait short questionnaire: Preliminary validation with Spanish adults. Span. J. Psychol..

[43] Goodman R, Meltzer H, Bailey V (2003). The strengths and difficulties questionnaire: A pilot study on the validity of the self-report version. Int. Rev. Psychiatry.

[44] Bevelander KE (2018). Youth’s social network structures and peer influences: Study protocol MyMovez project—phase I. BMC Public Health.

[45] Leary MR, Kelly KM, Cottrell CA, Schreindorfer LS (2013). Construct validity of the need to belong scale: Mapping the nomological network. J. Pers. Assess..

[46] Leary MR (1983). A brief version of the fear of negative evaluation scale. Personal. Soc. Psychol. Bull..

[47] Collins KA, Westra HA, Dozois DJA, Stewart SH (2005). The validity of the brief version of the fear of negative evaluation scale. J. Anxiety Disord..

[48] Graziano WG, Jensen-Campbell LA, Finch JF (1997). The self as a mediator between personality and adjustment. J. Pers. Soc. Psychol..

[49] Fabes RA, Carlo G, Kupanoff K, Laible D (1999). Early adolescence and prosocial/moral behavior I. J. Early Adolesc..

[50] Branje SJT, van Lieshout CFM, Gerris JRM (2007). Big Five personality development in adolescence and adulthood. Eur. J. Pers..

[51] Klimstra TA, Hale WW, Raaijmakers QAW, Branje SJT, Meeus WHJ (2009). Maturation of personality in adolescence. J. Pers. Soc. Psychol..

[52] Vecchione M, Alessandri G, Barbaranelli C, Caprara G (2012). Gender differences in the big five personality development: A longitudinal investigation from late adolescence to emerging adulthood. Pers. Individ. Dif..

[53] Luengo-Kanacri BP, Pastorelli C, Eisenberg N, Zuffianò A, Caprara GV (2013). The development of prosociality from adolescence to early adulthood: The role of effortful control. J. Pers..

[54] Van der Graaff J, Carlo G, Crocetti E, Koot HM, Branje S (2018). Prosocial behavior in adolescence: Gender differences in development and links with empathy. J. Youth Adolesc..

[55] Uliaszek AA (2010). The role of neuroticism and extraversion in the stress–anxiety and stress–depression relationships. Anxiety Stress Coping.

[56] Klimstra TA, Crocetti E, Hale WW, Fermani A, Meeus WHJ (2011). Big Five personality dimensions in Italian and Dutch adolescents: A cross-cultural comparison of mean-levels, sex differences, and associations with internalizing symptoms. J. Res. Pers..

[57] Iimura S, Taku K (2018). Gender differences in relationship between resilience and big five personality traits in japanese adolescents. Psychol. Rep..

[58] García-Lopez LJ, Olivares J, Hidalgo MD, Beidel DC, Turner SM (2001). Psychometric properties of the social phobia and anxiety inventory, the Social Anxiety Scale for Adolescents, the Fear of Negative Evaluation Scale, and the Social Avoidance and Distress Scale in an adolescent Spanish-speaking sample. J. Psychopathol. Behav. Assess..

[59] Duke D, Krishnan M, Faith M, Storch EA (2006). The psychometric properties of the Brief Fear of Negative Evaluation Scale. J. Anxiety Disord..

[60] Geukens F (2020). Changes in adolescent loneliness and concomitant changes in fear of negative evaluation and self-esteem. Int. J. Behav. Dev..

[61] Compton SN, Nelson AH, March JS (2000). Social phobia and separation anxiety symptoms in community and clinical samples of children and adolescents. J. Am. Acad. Child Adolesc. Psychiatry.

[62] Ingles CJ, La Greca AM, Marzo JC, Garcia-Lopez LJ, Garcia-Fernandez JM (2010). Social Anxiety Scale for Adolescents: Factorial invariance and latent mean differences across gender and age in Spanish adolescents. J. Anxiety Disord..

[63] Donnellan MB, Trzesniewski KH, Robins RW, Chamorro-Premuzic T, von Stumm S, Furnham A (2013). Self-esteem: Enduring issues and controversies. The Wiley-Blackwell Handbook of Individual Differences.

[64] London B, Downey G, Bonica C, Paltin I (2007). Social causes and consequences of rejection sensitivity. J. Res. Adolesc..

[65] Shi B, Dai DY, Lu Y (2016). Openness to experience as a moderator of the relationship between intelligence and creative thinking: A study of chinese children in urban and rural areas. Front. Psychol..

[66] Leibovich N, Schmid V, Calero A (2018). The need to belong (NB) in adolescence: Adaptation of a scale for its assessment. Psychol. Behav. Sci. Int. J..

[67] McCoy SS, Dimler LM, Samuels DV, Natsuaki MN (2019). Adolescent susceptibility to deviant peer pressure: Does gender matter?. Adolesc. Res. Rev..

[68] Closson LM (2009). Status and gender differences in early adolescents’ descriptions of popularity. Soc. Dev..

[69] Iwamoto DK, Smiler AP (2013). Alcohol makes you macho and helps you make friends: The role of masculine norms and peer pressure in adolescent boys’ and girls’ alcohol use. Subst. Use Misuse.

[70] Schulte MT, Ramo D, Brown SA (2009). Gender differences in factors influencing alcohol use and drinking progression among adolescents. Clin. Psychol. Rev..

[71] Carter M (2014). Gender socialization and identity theory. Soc. Sci..

[72] Rose AJ, Rudolph KD (2006). A review of sex differences in peer relationship processes: Potential trade-offs for the emotional and behavioral development of girls and boys. Psychol. Bull..

[73] Jarvinen DW, Nicholls JG (1996). Adolescents’ social goals, beliefs about the causes of social success, and satisfaction in peer relations. Dev. Psychol..

[74] Abele AE (2016). Facets of the fundamental content dimensions: Agency with competence and assertiveness—communion with warmth and morality. Front. Psychol..

[75] Hill, J. P. & Lynch, M. E. The intensification of gender-related role expectations during early adolescence. In *Girls at Puberty: Biological and Psychosocial Perspectives* (eds Brooks-Gunn, J. & Petersen, A. C.) 201–228 (Springer US, 1983). 10.1007/978-1-4899-0354-9_10.

[76] Korlat S (2021). Gender role identity and gender intensification: Agency and communion in adolescents’ spontaneous self-descriptions. Eur. J. Dev. Psychol..

[77] Chein J, Albert D, O’Brien L, Uckert K, Steinberg L (2011). Peers increase adolescent risk taking by enhancing activity in the brain’s reward circuitry. Dev. Sci..

[78] Somerville LH (2019). Dissecting “peer presence” and “decisions” to deepen understanding of peer influence on adolescent risky choice. Child Dev..

[79] Ciranka S, van den Bos W (2019). Social influence in adolescent decision-making: A formal framework. Front. Psychol..

[80] van Hoorn J, van Dijk E, Meuwese R, Rieffe C, Crone EA (2016). Peer influence on prosocial behavior in adolescence. J. Res. Adolesc..

[81] Byrnes JP, Miller DC, Schafer WD (1999). Gender differences in risk taking: A meta-analysis. Psychol. Bull..

[82] Telzer EH, van Hoorn J, Rogers CR, Do KT (2018). Social influence on positive youth development: A developmental neuroscience perspective. Adv. Child Dev. Behav..

[83] Steinberg L (2008). A social neuroscience perspective on adolescent risk-taking. Dev. Rev..

[84] Telzer EH (2016). Dopaminergic reward sensitivity can promote adolescent health: A new perspective on the mechanism of ventral striatum activation. Dev. Cogn. Neurosci..

[85] Albert D, Chein J, Steinberg L (2013). The teenage brain. Curr. Dir. Psychol. Sci..

[86] Foulkes L, Blakemore S-J (2016). Is there heightened sensitivity to social reward in adolescence?. Curr. Opin. Neurobiol..

[87] Alarcón G, Pfeifer JH, Fair DA, Nagel BJ (2018). Adolescent gender differences in cognitive control performance and functional connectivity between default mode and fronto-parietal networks within a self-referential context. Front. Behav. Neurosci..

