# Supplementary material for: The personality and cognitive traits associated with adolescents’ sensitivity to social norms
Source: Sci Rep. 2022 Sep 9;12:15247. doi: 10.1038/s41598-022-18829-x (PMC9463150; doi:10.1038/s41598-022-18829-x)
Supplement: Supplementary file 1 — Supplementary Information. [file 41598_2022_18829_MOESM1_ESM.docx]

Supplementary File 1: Appendix

Contents

[Part A: Procedures 2](#_Toc100924244)

[Part B: Data collection 3](#_Toc100924245)

[Part C: Game Theory Experiments 4](#_Toc100924246)

[Part D: Outcome Variable Transformation 10](#_Toc100924247)

[Part E: One-Way Analysis of Variance (ANOVA) 12](#_Toc100924248)

[Part F: Multilevel Mixed Effects Ordered Logistic Regression 14](#_Toc100924249)

[Part G: Multilevel Linear Mixed Effects Regression 17](#_Toc100924250)

List of Tables

[Table S1: Between group variance, ANOVA results (grouping variable: sensitivity to norms level) (males and females) 12](#_Toc100924251)

[Table S2: Personality and cognitive trait mean scores organised by sensitivity to norms level (males and females) 12](#_Toc100924252)

[Table S3: Between Group Variance, ANOVA Results (Grouping Variable: Sensitivity to Norms) (Male) 13](#_Toc100924253)

[Table S4: Between Group Variance, ANOVA Results (Grouping Variable: Sensitivity to Norms) (Female) 13](#_Toc100924254)

[Table S5: Univariate multilevel mixed effects ordered logistic regression results^1^ 14](#_Toc100924255)

[Table S6: Multivariate multilevel mixed effects ordered logistic regression results^1^ 14](#_Toc100924256)

[Table S7: Interactions with gender (univariate multilevel ordinal model)^1^ 15](#_Toc100924257)

[Table S8: Interactions with gender (multivariate multilevel ordinal model)^1^ 15](#_Toc100924258)

[Table S9: Univariate multilevel linear mixed effects model results^1^ 18](#_Toc100924259)

[Table S10: Multivariate multilevel linear mixed effects model results^1^ 18](#_Toc100924260)

# Part A: Procedures

A parental opt-out procedure was used with all participants providing their informed consent. Pupils who consented to participate took part in a baseline assessment consisting of a series of game theory experiments and completion of a self-report survey. Following the baseline assessment, each school took part in one of two previously tested smoking prevention interventions: ASSIST (N=3 schools in Northern Ireland [NI], N=3 Bogotá) or Dead Cool (N=3 NI, N=3 Bogotá) over a single school semester (approximately 10 weeks). Prior to implementation in Bogotá, all study materials (experiments, surveys, intervention materials) underwent a 'cultural adaptation' process, including translation into Spanish language and back translation, using a previously published framework. Participation in the study's experiments required a monetary payment to be made to each individual pupil. In NI the payment was made in cash, however due to Colombian ethical regulations the payment was made using gift cards to pupils in Bogotá.

# Part B: Data collection

The baseline assessment consisted of two separate sessions, held approximately one week apart, with each class in the school year group in each school. Sessions lasted approximately 50 minutes. Participating pupils completed the Game Theory experiments during the first session and the self-report survey during the second session. Experiments and surveys were collected on tablet computers using the platform Qualtrics (web-based platform in NI and offline version in Bogotá) (Qualtrics, Provo, Utah, USA). At the start of each session, participants were assured that any information provided would be treated as confidential. They were also instructed not to communicate with other participants and to direct any questions to a researcher. In NI, poster boards were used at computer stations to discourage communication between participants. In both countries, instructions were delivered onscreen with key portions read aloud by the experimenter. The experimenter read out introductory instructions at the start of the experiment. Pupils were invited to ask any questions. Dummy screens were inserted at the end of Parts 1 and 3 instructing pupils to wait until all of their classmates were ready to proceed to the next part so that instructions could be read together. Parts 2 and 3 were otherwise self-paced, and pupils were invited to raise their hand to have any further questions answered privately. The same procedures were used during the follow-up assessment.

# Part C: Game Theory Experiments

The game theory experiments consisted of a series of incentivized tasks which were based on published works in the field of behavioral economics, and designed by the original producers (Kimbrough, Krupka) and other experts in the field (Kumar, Ramalingam). There were four parts to the experiment: (1) a Rule-Following (RF) task measuring each individual participant's sensitivity to the effects of social norms; (2) a series of co-ordination games attempting to elicit *injunctive* social norms unrelated and related to smoking and vaping behaviors; (3) a series of co-ordination games attempting to elicit *descriptive* social norms related to smoking and vaping behaviors; (4) a willingness-to-pay task designed to measure each individual participant's support for cultivating anti-smoking norms. This study used the results from the RF task.

At the start of experimental sessions, participants were informed that they would receive a participation fee of £5.00 (NI; *COP* $5.000 in Bogotá), and that they could earn money in each part of the experiment (maximum £30 in NI, *COP* $50.000 in Bogotá) depending on the answers they provided and those provided by other pupils in their school year group. They were told that the researchers would determine their payment by performing two sets of randomizations for each part of the experiment: (1) to determine whether payment was based on answers provided at baseline or follow-up; (2) to determine which question of each part would result in payment.

The RF experiment consisted of an individual decision task measuring participants' preferences for following established rules and social norms, without peer interaction. The task instructs participants to follow an explicitly stated arbitrary rule when doing so provides them with no monetary benefits, but actually imposes explicit monetary costs directly proportional to the degree of rule-following. We employed the version of the RF task introduced by Kimbrough and Vostroknutov (2018). Specifically, participants were asked to sequentially allocate 50 balls across two buckets (one blue and one yellow). They were told that "The rule is to put the balls in the blue bucket". They were also informed that they would receive £0.05 (NI; *COP* $100 Bogotá) for every ball they put in the blue bucket and £0.10 (NI; *COP* $200 Bogotá) for every ball they put in the yellow bucket. Lastly, they were informed that they would be given five minutes to allocate the 50 balls between the two buckets and that any balls which were not allocated by the end of the five minutes were worth nothing.

No other information was provided. Therefore, assuming a participant allocated all 50 balls, the minimum amount that he/she could earn was £2.50 (NI; *COP* $5.000 Bogotá) if he/she followed the rule completely and allocated all 50 balls to the blue bucket. The maximum amount that could be earned was £5.00 (NI; *COP* $10.000 in Bogotá) if he/she completely ignored the rule and allocated all 50 balls to the yellow bucket. The central premise is that the more a participant cares intrinsically about rule-following the more willing he/she will be to incur the costs of doing so.

Every participant allocated all 50 balls to a bucket during the baseline and follow-up experiments during the full phase. There were several changes made to the experimental protocol after baseline was completed in the first pilot school in Northern Ireland. The first version of the experiment included a forced waiting time for the RF task of seven minutes, the idea being to make sure that all pupils proceeded to the second part together. Subsequently it was decided to reduce the time allocated for the RF task from seven minutes to five minutes, to remove the forced waiting time and to insert dummy screens informing participants when to wait for further instructions from the experimenter. Data from MECHANISMS pilot schools are not included in the current paper.


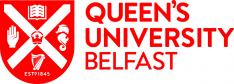


**Experimental Instructions**

**General information**

This is a study about decision-making. You will be paid a fee of £5 for taking part, as outlined below. In addition, you may receive some extra money based on your choices and the choices made by others during the study.

If you have any questions during the session, please raise your hand and wait for a researcher to come to you.  Please do not talk or try to communicate with other participants during the experiment.  It is important that everyone taking part makes his or her own decisions.

This is an on-going study, which has received funding from the UK Medical Research Council to cover all current and future costs. You can be certain that all participants who complete the study will be paid as described in the instructions. If you have any concerns, please contact:

**Dr. Ruth Hunter**
Centre for Public Health/UKCRC Centre of Excellence for Public Health (NI)
School of Medicine, Dentistry and Biomedical Sciences
Institute of Clinical Science B, Royal Victoria Hospital
Grosvenor Road, Belfast, BT 12 6BJ
E-mail: ruth.hunter@qub.ac.uk; 
Tel: +44 (0) 28 90978944
 
**There are four parts to today’s study.**

**You can earn money in each part.**

Your earnings from today will **not** be paid to you today. We will come back to your school at the end of the program in ten weeks’ time. At that time, we would like you to participate in another study. There will be four parts to that study, and you can earn money in each part of that study too.

After you have participated in the study at the end of the program we will determine for each part whether you receive earnings from today or from the study at the end of the program. For each part, we will toss a coin to determine this. We will record your choices in both today’s study and the study at the end of the program. You will be able to review your choices from both experiments when you learn your payment, if you wish.

**Part 1**

In Part 1 of this study, you will decide how to allocate 50 balls between two buckets. Your task is to put each of the balls, one-by-one, into one of the two buckets: the blue bucket or the yellow bucket. The balls will appear to the left-hand side of your screen, and you can allocate each ball by clicking and dragging it to the bucket of your choice. For each ball you put in the blue bucket, you will receive 5 pence, and for each ball you put in the yellow bucket, you will receive 10 pence.

The rule is to put the balls in the blue bucket.

Once the experiment begins, you will have 5 minutes to put the balls into the buckets. When you are finished, please click on the next button and wait quietly for further instructions from the experimenter. Any balls that have not been placed in a bucket at the end of the 5 minutes are worth nothing. Your earnings from Part 1 will be based on your decisions: it is the sum of earnings from the blue and yellow buckets.

This is the end of the instructions for Part 1. If you have any questions, please raise your hand and a researcher will answer them privately. Otherwise, please wait quietly until all of your classmates are ready and click on the next button to begin the experiment.


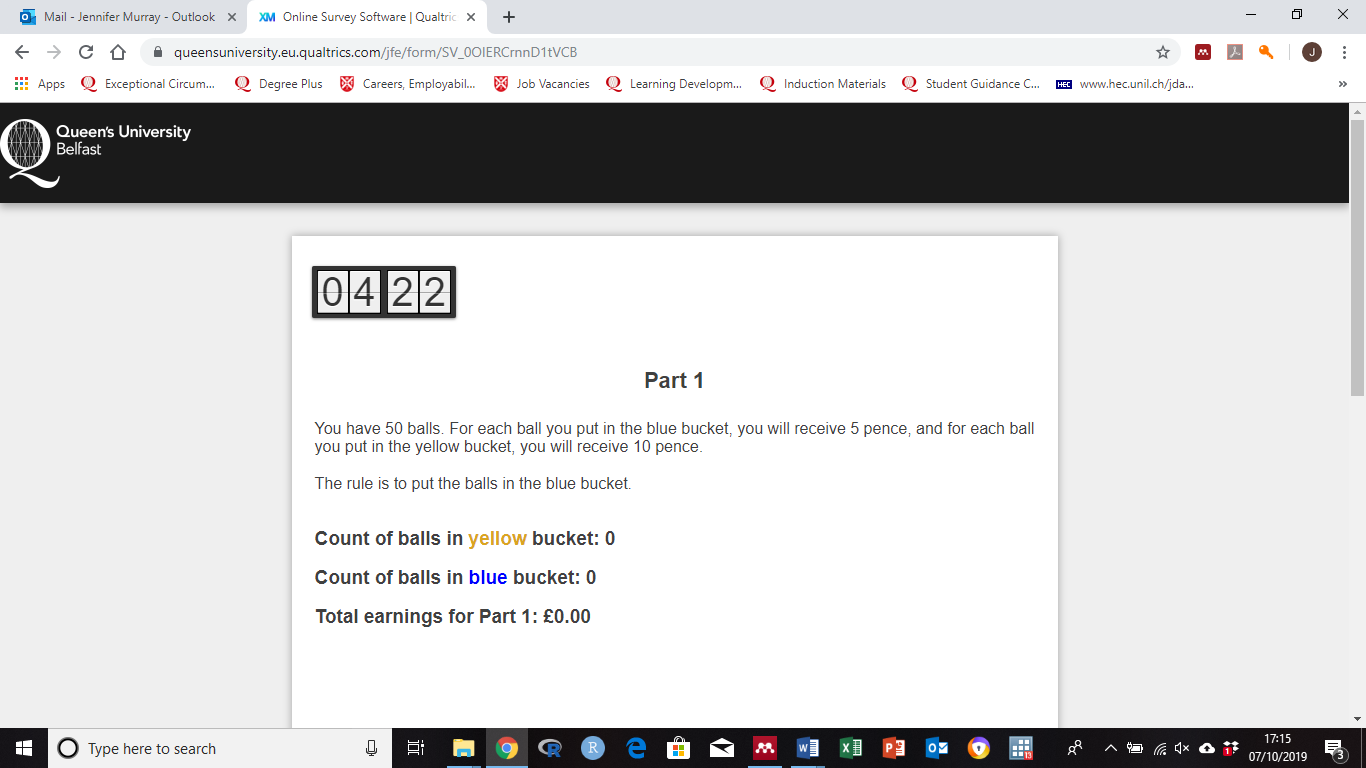


**The 50 balls can be re-located individually to either the blue or yellow bucket by mouse click and drag-and-drop.**

**Updated as balls are dragged in or out of the blue bucket.**

**Timer indicating five-minute count-down for completing Rule-Following task.**


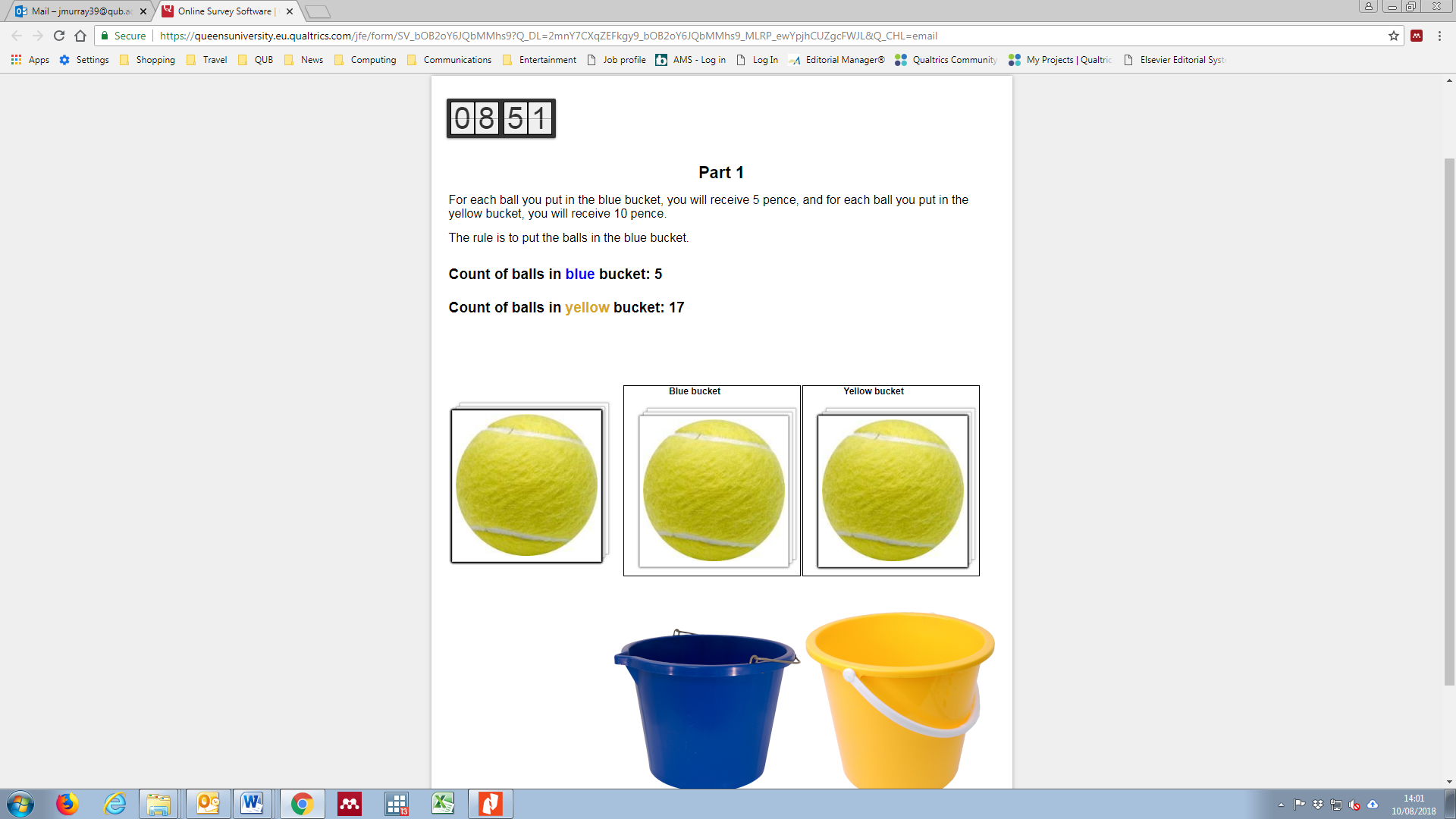


**Updated as balls are dragged in or out of the yellow bucket.**

**N.B. Participants were randomised to this version of the experiment or to a version that had the buckets in reverse order to overcome any potential bias due to positioning of buckets.**

# Part D: Outcome Variable Transformation

***Description of the current variable***

As shown in figure S1. The distribution of rule-following propensity is skewed slightly towards full rule-following (50, n=383/1,216). However, the distribution also has point masses at full rule-breaking (0, n=151/1,216) and neutral (25, n=169/1,216).

Figure S1: Rule-following propensity (number of balls in blue bucket). Number of observations: 1,216

**n** = 1,216

**Mean**: 31.00

**SD**: 17.98

**SE**: 0.516

**Median**: 31.00

**Shapiro-Wilk W**: 0.966 (p < 0.001)

**Skewness** = -.452 (p < 0.001)

**Kurtosis** = 1.894 (p < 0.001)

***Recoding scheme***

In line with Kimbrough and Vostroknutov (2018) we classified “full rule-following” individuals as those who allocated 50 balls to the blue bucket and “full rule-breaking” individuals as those who allocated 0 balls to the blue bucket. Our approach deviates from that of Kimbrough and Vostroknutov with the inclusion of 3 new levels: “Prefer rule-breaking” (1-24); “Neutral” (25); and “Prefer rule-following” (26-49).

# Part E: One-Way Analysis of Variance (ANOVA)

The tables below show the results of one-way analysis of variance (ANOVA) and mean scores for each of the personality and cognitive traits for adolescents according to which level of norms sensitivity they were classified as.

Table S1: Between group variance, ANOVA results (grouping variable: sensitivity to norms level) (males and females)

| Independent variable | SS | df | MS | F | Prob > F | Prob>chi2^1^ |
| --- | --- | --- | --- | --- | --- | --- |
| Openness | 4.7418 | 4 | 1.18545 | 2.56 | 0.037 | 0.554 |
| Extraversion | 4.397333 | 4 | 1.099333 | 2.05 | 0.086 | 0.102 |
| Agreeableness | 5.011197 | 4 | 1.252799 | 2.99 | 0.018 | 0.699 |
| Conscientiousness | 3.113756 | 4 | 0.778439 | 1.83 | 0.120 | 0.009 |
| Emotional Stability | 0.830355 | 4 | 0.207589 | 0.36 | 0.834 | 0.017 |
| Prosociality | 34.28067 | 4 | 8.570168 | 1.91 | 0.106 | 0.061 |
| Need to belong | 0.693018 | 4 | 0.173254 | 0.44 | 0.780 | 0.140 |
| Fear of negative evaluation | 3.865893 | 4 | 0.966473 | 2.39 | 0.049 | 0.000 |
| ^1^ Bartlett's test for equal variances | | | | | | |

| Legend: |
| --- |
| SS = Sum-of-squares |
| df = Degrees of freedom |
| MS = Mean squares |
| F = F-ratio |
| Prob > F = p-value  Prob > chi2 = Bartlett’s test p-value |

Table S2: Personality and cognitive trait mean scores organised by sensitivity to norms level (males and females)

|  |  | **Sensitivity to norms** | | | | |
| --- | --- | --- | --- | --- | --- | --- |
|  | **Independent Variable** | 1 | 2 | 3 | 4 | 5 |
| **Mean scores** | Openness* | 2.44 | 2.53 | 2.56 | 2.62 | 2.63 |
|  | Extraversion | 2.51 | 2.59 | 2.62 | 2.69 | 2.69 |
|  | Agreeableness* | 2.44 | 2.51 | 2.65 | 2.60 | 2.63 |
|  | Conscientiousness | 2.25 | 2.28 | 2.36 | 2.36 | 2.40 |
|  | Emotional Stability | 2.06 | 1.97 | 2.04 | 2.01 | 2.00 |
|  | Prosociality | 7.32 | 7.65 | 7.73 | 7.73 | 7.88 |
|  | Need to belong | 2.95 | 2.96 | 3.00 | 2.95 | 2.93 |
|  | Fear of negative evaluation* | 2.87 | 2.70 | 2.84 | 2.76 | 2.73 |
| ***** p ≤ 0.05 in ANOVA | | | | | | |

| Legend: |
| --- |
| 1 = Full rule-breaking |
| 2 = Prefer rule-breaking |
| 3 = Neutral |
| 4 = Prefer rule-following |
| 5 = Full rule-following |

Tables S3 and S4 show the results of ANOVA tests conducted after disaggregating the data into male and female subgroups.

Table S3: Between Group Variance, ANOVA Results (Grouping Variable: Sensitivity to Norms) (Male)

| Independent variable | SS | df | MS | F | Prob > F | Prob>chi2^1^ |
| --- | --- | --- | --- | --- | --- | --- |
| Openness | 1.936634 | 4 | 0.484158 | 0.94 | 0.441 | 0.885 |
| Extraversion | 1.407842 | 4 | 0.35196 | 0.66 | 0.620 | 0.039 |
| Agreeableness | 4.115732 | 4 | 1.028933 | 2.56 | 0.038 | 0.630 |
| Conscientiousness | 3.018634 | 4 | 0.754659 | 1.83 | 0.122 | 0.000 |
| Emotional Stability | 4.618911 | 4 | 1.154728 | 2.36 | 0.052 | 0.050 |
| Prosociality | 10.22125 | 4 | 2.555313 | 0.50 | 0.733 | 0.965 |
| Need to belong | 0.968089 | 4 | 0.242022 | 0.64 | 0.634 | 0.177 |
| Fear of negative evaluation | 3.237325 | 4 | 0.809331 | 2.44 | 0.046 | 0.000 |
| ^1^ Bartlett's test for equal variances | | | | | | |

Table S4: Between Group Variance, ANOVA Results (Grouping Variable: Sensitivity to Norms) (Female)

| Independent variable | SS | df | MS | F | Prob > F | Prob>chi2^1^ |
| --- | --- | --- | --- | --- | --- | --- |
| Openness | 0.767808 | 4 | 0.191952 | 0.47 | 0.759 | 0.865 |
| Extraversion | 2.942461 | 4 | 0.735615 | 1.35 | 0.251 | 0.698 |
| Agreeableness | 1.334122 | 4 | 0.33353 | 0.78 | 0.540 | 0.105 |
| Conscientiousness | 0.549624 | 4 | 0.137406 | 0.31 | 0.870 | 0.609 |
| Emotional Stability | 1.297747 | 4 | 0.324437 | 0.55 | 0.698 | 0.257 |
| Prosociality | 13.83144 | 4 | 3.45786 | 0.94 | 0.440 | 0.017 |
| Need to belong | 1.31278 | 4 | 0.328195 | 0.80 | 0.523 | 0.216 |
| Fear of negative evaluation | 4.520906 | 4 | 1.130227 | 2.42 | 0.047 | 0.004 |
| ^1^ Bartlett's test for equal variances | | | | | | |

# Part F: Multilevel Mixed Effects Ordered Logistic Regression

Tables S5 and S6 show the results from the multilevel mixed effects ordered logistic regression analyses that was conducted treating the outcome variable (sensitivity to norms) as ordinal.

Table S5: Univariate multilevel mixed effects ordered logistic regression results^1^

|  | Overall | | Males | | Females | |
| --- | --- | --- | --- | --- | --- | --- |
| **Independent Variables** | Coef. | 95% CI | Coef. | 95% CI | Coef. | 95% CI |
| **Demographics** |  |  |  |  |  |  |
| Gender | 0.46* | (0.24-0.67) |  |  |  |  |
| Age | -0.04 | (-0.17-0.09) | 0.00 | (-0.19-0.19) | -0.01 | (-0.21-0.18) |
| Ethnicity | -0.02 | (-0.36-0.31) | 0.00 | (-0.47-0.47) | 0.03 | (-0.46-0.52) |
| Single Parent | -0.03 | (-0.26-0.21) | 0.11 | (-0.23-0.46) | -0.17 | (-0.5-0.15) |
| **Big Five Personality Traits** |  |  |  |  |  |  |
| Openness | 0.19* | (0.03-0.36) | 0.17 | (-0.05-0.39) | 0.17 | (-0.07-0.42) |
| Extraversion | 0.18* | (0.03-0.33) | 0.15 | (-0.07-0.37) | 0.18 | (-0.03-0.38) |
| Agreeableness | 0.17 | (0-0.34) | 0.28* | (0.03-0.53) | 0.03 | (-0.21-0.26) |
| Conscientiousness | 0.16 | (0-0.33) | 0.30* | (0.05-0.54) | 0.02 | (-0.21-0.26) |
| Emotional Stability | -0.05 | (-0.2-0.09) | 0.21 | (-0.02-0.44) | -0.15 | (-0.36-0.05) |
| **Other Cognitive Traits** |  |  |  |  |  |  |
| Prosociality | 0.05 | (0-0.1) | 0.04 | (-0.03-0.11) | 0.02 | (-0.06-0.1) |
| Need to belong | -0.08 | (-0.25-0.09) | -0.22 | (-0.48-0.04) | -0.04 | (-0.28-0.2) |
| Fear of negative evaluation | -0.15 | (-0.33-0.02) | -0.51* | (-0.8--0.22) | 0.07 | (-0.16-0.3) |
| * p ≤ 0.05  ^1^ Dependent variable: sensitivity to norms level (computed as ordinal) | | | | | | |

Table S6: Multivariate multilevel mixed effects ordered logistic regression results^1^

|  | Overall | | Males | | Females | |
| --- | --- | --- | --- | --- | --- | --- |
| **Independent Variables** | Coef. | 95% CI | Coef. | 95% CI | Coef. | 95% CI |
| **Demographics** |  |  |  |  |  |  |
| Gender | 0.42* | (0.18-0.66) |  |  |  |  |
| Age | -0.01 | (-0.15-0.13) | -0.01 | (-0.21-0.18) | -0.05 | (-0.26-0.15) |
| Ethnicity | -0.09 | (-0.45-0.28) | -0.15 | (-0.65-0.35) | -0.06 | (-0.60-0.48) |
| Single Parent | -0.10 | (-0.34-0.15) | 0.03 | (-0.33-0.39) | -0.33 | (-0.67-0.02) |
| **Big Five Personality Traits** |  |  |  |  |  |  |
| Openness | 0.03 | (-0.12-0.18) | 0.04 | (-0.17-0.24) | 0.02 | (-0.19-0.24) |
| Extraversion | 0.09 | (-0.05-0.23) | -0.01 | (-0.23-0.21) | 0.16 | (-0.02-0.35) |
| Agreeableness | 0.04 | (-0.11-0.20) | 0.12 | (-0.11-0.36) | -0.02 | (-0.24-0.19) |
| Conscientiousness | 0.08 | (-0.07-0.23) | 0.12 | (-0.10-0.33) | 0.05 | (-0.15-0.26) |
| Emotional Stability | -0.11 | (-0.26-0.03) | -0.04 | (-0.26-0.18) | -0.21* | (-0.41--0.01) |
| **Other Cognitive Traits** |  |  |  |  |  |  |
| Prosociality | 0.00 | (-0.13-0.14) | 0.06 | (-0.13-0.24) | 0.02 | (-0.18-0.23) |
| Need to belong | -0.12 | (-0.26-0.02) | -0.21* | (-0.41-0.00) | -0.10 | (-0.30-0.10) |
| Fear of negative evaluation | -0.07 | (-0.22-0.08) | -0.21 | (-0.44-0.02) | 0.04 | (-0.16-0.25) |
| * p ≤ 0.05  ^1^ Dependent variable: sensitivity to norms level (computed as ordinal) | | | | | | |

Tables S7 and S8 show univariate and multivariate interactions for gender with each independent variable used in the multilevel mixed effects ordered logistic regression models.

Table S7: Interactions with gender (univariate multilevel ordinal model)^1^

|  | Overall | | |
| --- | --- | --- | --- |
| **Independent Variables** | Coef. | 95% CI | p > z |
| **Demographics** |  |  |  |
| Age x Gender | 0.02 | (-0.24-0.27) | 0.904 |
| Ethnicity x Gender | 0.01 | (-0.66-0.68) | 0.970 |
| Single Parent x Gender | -0.30 | (-0.76-0.16) | 0.207 |
| **Big Five Personality Traits** |  |  |  |
| Openness x Gender | -0.07 | (-0.39-0.25) | 0.649 |
| Extraversion x Gender | -0.03 | (-0.32-0.27) | 0.859 |
| Agreeableness x Gender | -0.31 | (-0.65-0.03) | 0.070 |
| Conscientiousness x Gender | -0.34 | (-0.67-0.00) | 0.050 |
| Emotional Stability x Gender | -0.41 | (-0.72--0.11) | 0.008 |
| **Other Cognitive Traits** |  |  |  |
| Prosociality x Gender | -0.04 | (-0.14-0.06) | 0.469 |
| Need to belong x Gender | 0.25 | (-0.1-0.6) | 0.162 |
| Fear of negative evaluation x Gender | 0.59 | (0.22-0.96) | 0.002 |
| ^1^ Dependent variable: sensitivity to norms level (computed as ordinal) | | | |

Table S8: Interactions with gender (multivariate multilevel ordinal model)^1^

|  | Overall | | |
| --- | --- | --- | --- |
| **Independent Variables** | Coef. | 95% CI | p > z |
| **Demographics** |  |  |  |
| Age x Gender | 0.04 | (-0.23-0.31) | 0.773 |
| Ethnicity x Gender | 0.16 | (-0.55-0.87) | 0.656 |
| Single Parent x Gender | -0.36 | (-0.85-0.12) | 0.142 |
| **Big Five Personality Traits** |  |  |  |
| Openness x Gender | -0.09 | (-0.32-0.14) | 0.431 |
| Extraversion x Gender | -0.02 | (-0.26-0.21) | 0.852 |
| Agreeableness x Gender | -0.22 | (-0.45-0.01) | 0.059 |
| Conscientiousness x Gender | -0.2 | (-0.43-0.02) | 0.079 |
| Emotional Stability x Gender | -0.34 | (-0.58--0.1) | 0.005 |
| **Other Cognitive Traits** |  |  |  |
| Prosociality x Gender | -0.08 | (-0.31-0.15) | 0.493 |
| Need to belong x Gender | 0.28 | (0.05-0.52) | 0.018 |
| Fear of negative evaluation x Gender | 0.36 | (0.11-0.61) | 0.005 |
| ^1^ Dependent variable: sensitivity to norms level (computed as ordinal) | | | |

# Part G: Multilevel Linear Mixed Effects Regression

Tables S9 and S10 show the results from the multilevel linear mixed effects models treating the outcome variable (sensitivity to norms) as continuous.

The first series of univariate multilevel linear mixed effect models assessed the relationship between the independent variables and individual sensitivity to norms. In the second step, a multivariate multilevel linear mixed effects model was computed by simultaneously entering all the independent variables into the model. These models were run for the whole sample as well as the male and female samples separately.

Results from the univariate multilevel linear mixed effects models are shown in Table S9. Gender was significantly associated with sensitivity to norms (β=4.84, z=4.89, p<0.001) with females scoring higher than males. Of the Big 5 personality traits, significant positive relationships were found for openness (β=2.04, z=2.73, p=0.006), extraversion (β=1.71, z=2.48, p=0.013), and conscientiousness (β=1.55, z=1.97, p=0.049).

In the male subgroup, significant positive associations were found for agreeableness (β=2.82, z=2.33, p=0.020), conscientiousness (β=2.71, z=2.25, p=0.025), emotional stability (β=2.22, z=2.01, p=0.044) and sensitivity to norms. Fear of negative evaluation was negatively associated with sensitivity to norms in the male subgroup (β=-3.35, z=-2.56, p=0.011). Females who scored higher on the openness (β=2.22, z=2.15, p=0.031) and extraversion (β=1.96, z=2.22, p=0.026) scales were found to have a higher sensitivity to norms score in the univariate multilevel linear mixed effects model.

The results of the multivariate multilevel linear mixed effects model are shown in Table S10. After adjusting for all other independent variables in the overall sample, gender (β=4.67, z=4.27, p=0.000) and need to belong (β=-1.28, z=-1.98, p=0.048) were the only factors significantly associated with sensitivity to norms. No independent variables were significantly associated with sensitivity to norms in the male subgroup. In the female subgroup, living with a single parent was significantly associated with a lower score on the sensitivity to norms scale (β=-3.35, z=-2.28, p=0.023).

Table S9: Univariate multilevel linear mixed effects model results^1^

|  | Overall | | Males | | Females | |
| --- | --- | --- | --- | --- | --- | --- |
| **Independent Variables** | Coef. | 95% CI | Coef. | 95% CI | Coef. | 95% CI |
| **Demographics** |  |  |  |  |  |  |
| Gender | 4.84* | (2.90-6.78) |  |  |  |  |
| Age | -0.50 | (-1.74-0.74) | -0.02 | (-1.83-1.78) | -0.29 | (-1.98-1.39) |
| Ethnicity | -0.01 | (-3.22-3.19) | 0.01 | (-4.59-4.60) | 0.85 | (-3.50-5.21) |
| Single Parent | -0.78 | (-2.97-1.42) | 1.15 | (-2.16-4.45) | -2.05 | (-4.91-0.80) |
| **Big Five Personality Traits** |  |  |  |  |  |  |
| Openness | 2.04* | (0.58-3.51) | 1.70 | (-0.42-3.81) | 2.22* | (0.20-4.23) |
| Extraversion | 1.71* | (0.36-3.07) | 1.22 | (-0.86-3.30) | 1.96* | (0.23-3.70) |
| Agreeableness | 1.52 | (-0.02-3.07) | 2.82* | (0.44-5.19) | 0.15 | (-1.82-2.13) |
| Conscientiousness | 1.55* | (0.00-3.09) | 2.71* | (0.35-5.08) | 0.52 | (-1.44-2.48) |
| Emotional Stability | -0.40 | (-1.73-0.94) | 2.22* | (0.06-4.37) | -1.08 | (-2.77-0.60) |
| **Other Cognitive Traits** |  |  |  |  |  |  |
| Prosociality | 0.41 | (-0.06-0.88) | 0.48 | (-0.18-1.15) | -0.07 | (-0.75-0.61) |
| Need to belong | -0.57 | (-2.16-1.01) | -1.45 | (-3.92-1.02) | -0.98 | (-3.01-1.04) |
| Fear of negative evaluation | -0.98 | (-2.56-0.60) | -3.35* | (-5.92--0.78) | 0.26 | (-1.67-2.18) |
| * p ≤ 0.05  ^1^ Dependent variable: sensitivity to norms (computed as continuous) | | | | | | |

Table S10: Multivariate multilevel linear mixed effects model results^1^

|  | Overall | | Males | | Females | |
| --- | --- | --- | --- | --- | --- | --- |
| **Independent Variables** | Coef. | 95% CI | Coef. | 95% CI | Coef. | 95% CI |
| **Demographics** |  |  |  |  |  |  |
| Gender | 4.71* | (2.56-6.85) |  |  |  |  |
| Age | -0.29 | (-1.55-0.98) | 0.00 | (-1.88-1.88) | -0.91 | (-2.6-0.78) |
| Ethnicity | -0.66 | (-4.03-2.71) | -1.54 | (-6.41-3.33) | 0.27 | (-4.27-4.8) |
| Single Parent | -1.01 | (-3.27-1.26) | 0.60 | (-2.89-4.09) | -3.35* | (-6.23--0.47) |
| **Big Five Personality Traits** |  |  |  |  |  |  |
| Openness | 0.58 | (-0.72-1.89) | 0.72 | (-1.2-2.63) | 1.05 | (-0.69-2.79) |
| Extraversion | 0.83 | (-0.41-2.07) | -0.61 | (-2.68-1.45) | 1.45 | (-0.06-2.96) |
| Agreeableness | 0.23 | (-1.17-1.63) | 1.20 | (-1-3.4) | -0.44 | (-2.18-1.3) |
| Conscientiousness | 0.54 | (-0.78-1.85) | 0.74 | (-1.32-2.79) | 0.43 | (-1.23-2.09) |
| Emotional Stability | -0.70 | (-2.00-0.60) | 0.35 | (-1.71-2.41) | -1.58 | (-3.19-0.03) |
| **Other Cognitive Traits** |  |  |  |  |  |  |
| Prosociality | -0.04 | (-1.29-1.2) | 0.74 | (-1.03-2.52) | -0.34 | (-2.03-1.36) |
| Need to belong | -1.28* | (-2.55--0.01) | -1.86 | (-3.83-0.11) | -1.31 | (-2.94-0.32) |
| Fear of negative evaluation | -0.17 | (-1.5-1.15) | -0.90 | (-2.97-1.17) | 0.51 | (-1.16-2.18) |
| * p ≤ 0.05  ^1^ Dependent variable: sensitivity to norms (computed as continuous) | | | | | | |
